# Evaluating the role of levels of exposure to a task shared depression counselling intervention led by behavioural health counsellors: outcome and process evaluation

**DOI:** 10.1186/s13033-019-0299-2

**Published:** 2019-06-10

**Authors:** One Selohilwe, Arvin Bhana, Emily C. Garman, Inge Petersen

**Affiliations:** 1 Centre for Rural Health, School of Nursing and Public Health and School of Applied Human Sciences, University of KwaZulu-Natal, Durban, South Africa; 2 Health Systems Research Unit, South African Medical Research Council, Durban, South Africa; 3 Alan J Flisher Centre for Public Mental Health, Department of Psychiatry and Mental Health, University of Cape Town, Cape Town, South Africa

**Keywords:** Depression, Mental health, Psychosocial interventions, LMICs, Task sharing, Lay health workers

## Abstract

**Background:**

In the context of a large treatment gap for common mental disorders (CMDs) and shortage of mental health specialists in low- and middle-income countries, there is increasing evidence of the effectiveness of task sharing of counselling interventions to increase access to mental health care for CMDs at primary health care level. This study evaluated the relationship between levels of exposure to a task-shared counselling intervention and psychosocial outcomes (depression, functional disability, internalised stigma and social support) in chronic care service users with comorbid depression in South Africa guided by the Medical Research Council process evaluation framework. Implementation and participant-level factors that promote greater exposure were also investigated.

**Method:**

The study design was a cohort study comprising of 173 participants referred by primary health care nurses for the task-shared counselling intervention. The study site comprised four primary health care facilities in a sub-district of the Dr. Kenneth Kaunda district in the North West Province of South Africa. The participants were assessed for psychosocial outcomes at three time points: baseline, 3 months and at 12 months. The number of counselling sessions each participant was exposed to was collected for each participant. Linear regression models were used to test the influence of counselling exposure on each of the psychosocial variables between baseline and endline. In-depth qualitative interviews were conducted on 29 randomly selected participants, stratified according to exposure to counselling sessions, and analysed using framework analysis.

**Findings:**

Findings from the cohort study indicated a significant reduction in depression severity at 12 months. Internalised stigma and functional disability improved from baseline to endline. Participants receiving 5–8 sessions have the greatest reduction in PHQ9 scores from baseline to endline (β = − 2.46, 95% CI − 5.06 to 0.15) compared to those with 0 sessions (β = − 0.51, 95% CI − 3.62 to 2.60, *p* = 0.064). The WHODAS scores decreased significantly more from baseline to endline among those who received 5–8 sessions (β = − 10.73, 95% CI − 19.86 to 1.59) compared to those with 0 sessions (β = 2.25, 95% CI − 8.65 to 13.14, *p *= 0.021). No significant differences as a function of levels of counselling exposure from baseline to endline was observed for OSS-3 scores. An improvement in ISMI scores from 1–4 sessions to 5–8 sessions was found (β = − 4.05, 95% CI − 7.30 to − 0.80, *p *= 0.015). The qualitative process evaluation indicated that the service was acceptable and accessible; but that session attendance was hindered by women’s’ caregiving burden, poor counsellor attributes and poor referral processes.

**Conclusion:**

Exposure to a greater number of sessions (5–8 sessions) was found to optimize functional ability, reduce stigma, and potentially reduce depression symptoms. In order to enhance session attendance, lay counsellor delivered psychosocial interventions need to pay attention to (i) counsellor selection criteria, particularly person-centred care qualities; and (ii) strengthening referral processes in contexts where mental health literacy is low.

## Introduction

Non communicable diseases (NCDs) have become the leading cause of global mortality, accounting for 68% (38 million) of deaths in 2012 [1]. Cardiovascular diseases, cancer, chronic respiratory diseases and cancer were responsible for 82% of these deaths. While infectious disease deaths are expected to decrease, non-communicable diseases are projected to increase by 47% by the year 2030 [1].

In South Africa, NCDs accounted for 29% of mortality in 2009 [2], a figure which increased to 55.5% in 2015 [3]. Diabetes, cerebrovascular diseases and other forms of diseases are now outcompeting HIV as the leading cause of death [4]. With the roll out of antiretroviral therapy (ART), HIV/AIDS has transitioned to a chronic condition and HIV-infected people are living longer, leading to an increase of service users on ART [5–7]. Statistics show the number of people in treatment has increased from approximately 50,000 in 2004 to 3,389,000 in 2015 [6]. In order to achieve universal access to HIV care and treatment, necessary as a pre-condition for achieving national viral load suppression, South Africa has adopted the global targets to identify 90% of people living with HIV, to have 90% of people identified on ART treatment and have 90% of people receiving ART to be virally suppressed to be achieved by 2020 [6, 7].

These targets, in conjunction with the rising burden of chronic NCDs, are straining South Africa’s health-care system [5, 8]. HIV and other chronic conditions also often co-exist [5]. In this respect, ART has been linked to increased risk of diabetes, dyslipidaemia, and myocardial infarction [5, 8]. Common mental disorders (CMDs), including depression and anxiety disorders, also often co-occur with physical illnesses such as hypertension, HIV, and diabetes, further complicating the fight against the rising burden of chronic NCDs. Pathways leading to comorbidity of mental disorders and NCDs are complex and bidirectional, with a mutually reinforcing relationship [8–10]. Research shows that people with depression comorbid with HIV are 55% less likely to adhere to medication when compared to HIV positive people who are not depressed, leading to poor health outcomes [11]. Furthermore, people diagnosed with HIV are twice more likely to get depressed than the general population [10]. This leads to increased costs to the health care system due to greater service utilization. Untreated depression is also a risk factor for death related to cardiovascular disease and stroke. Studies show that comorbid depression triples the likelihood of death in service users with myocardial infarct [12].

Although treatment exists, a large proportion of people living with mental disorders do not receive the treatment they need and the majority of these people are found in low- and middle-income countries (LMICs) [13]. This is because of competing health priorities for LMICs which are focused on dealing with infectious diseases and reproductive, maternal, and child health [14]. Furthermore, resources and expertise for mental health care are extremely limited, creating a huge gap between the need for care and the ability to treat [13, 15]. Research shows between 76 and 84% of people who need services for severe mental disorders in LMICs do not receive them [13, 16]. South Africa is no exception, with an estimated treatment gap of 75% for CMDs [17]. Closing the treatment gap will assist to reduce associated disability-adjusted life-years (DALYs) as well as alleviate the socioeconomic impact mental health has on individuals and their societies [18]. Chisholm et al. [19] argue that, to see a significant reduction in the burden, there needs to be a substantial increase in treatment coverage.

In response to the need to close the treatment gap, the World Health Organisation (WHO) has laid out 10 recommendations which include mental health treatment being accessible at primary care, increasing and improving training of mental health professionals and establishing national mental health programmes [20]. South Africa responded by committing to providing equitable access to mental health care and scaling up of decentralized integrated primary mental health service as contained in the first post-democracy National Mental Health Policy Framework and Action Plan (2013–2020) [21].

Primary health care provides universal access to essential care in communities, providing a platform to facilitate increased access to mental health care [22]; as well as providing an opportunity to implement preventative measures. In South Africa, primary health care facilities are typically the first point of access to health care and are situated in communities close to the people. Integrating mental health care into primary setting increases the chance for better health outcomes overall as individuals will be treated holistically as opposed to focusing on a single condition.

The delivery of mental health care in South Africa is impeded by a shortage of specialist human resources in primary health care settings in particular [10]. General health workers, such as lay health counsellors in the South African context present a potential resource for bridging the treatment gap through embracing task-sharing. Research has shown that the use of adequately supervised lay health workers to provide counselling in resource-constrained settings can yield desirable outcomes, enhancing health care capacity and extending services to more service users [11, 23]. The Programme for Improving Mental health carE [13] in South Africa (PRIME-SA) developed and evaluated a model for integrating mental health care within the nascent integrated chronic care delivery system. As part of this, PRIME-SA developed a collaborative care model for depression which includes counsellor-led depression counselling under the supervision of psychologists [13, 24].

In the PRIME-SA collaborative care model, nurses identify service users with depression, and refer users with moderate to severe symptoms to doctors for initiation of anti-depressant medication and users with mild to moderate symptoms to the lay counsellors for structured manualized depression counselling under the supervision of a psychologist.

The lay counselling intervention draws on cognitive behavioural therapy (CBT) techniques, particularly problem-solving and behavioural activation. These have been shown to produce favourable outcomes within a task-shared approach for people with depressive symptoms, and is recommended by the WHO’s Mental Health Gap Action Programme (mhGAP) [25, 26]. When delivered within groups, these techniques have the potential to promote resilience and harness the power of social support [10, 27].

A non-randomly assigned comparison group cohort study found that patients referred for care within the collaborative stepped care model demonstrated significant clinical improvements compared to those who were not referred [28]. Guided by the Medical Research Council (MRC) framework for the evaluation of complex interventions [29], the aim of this paper was to evaluate the relationship between levels of exposure to the task-shared counselling intervention component of the collaborative care model, and psychosocial outcomes (depression, functional disability and social support) in chronic care service users with comorbid depression; as well as understand implementation and participant-level mechanisms of impact that promoted greater exposure to the intervention guided by the MRC framework for process evaluation [29].

## Methods

### Study site

The study site was made up of four primary health care facilities in the Kanana township, an urban area, in the Matlosana sub-district of Dr. Kenneth Kaunda district in the North West Province. These clinics serviced an estimated population of 78,400 people [30]. The clinics are staffed by nurses and rotational medical officers. A primary health care (PHC) psychologist also serves these four clinics as well as 13 other facilities in the Matlosana sub-district.

### Intervention description

**Fig. 1:**
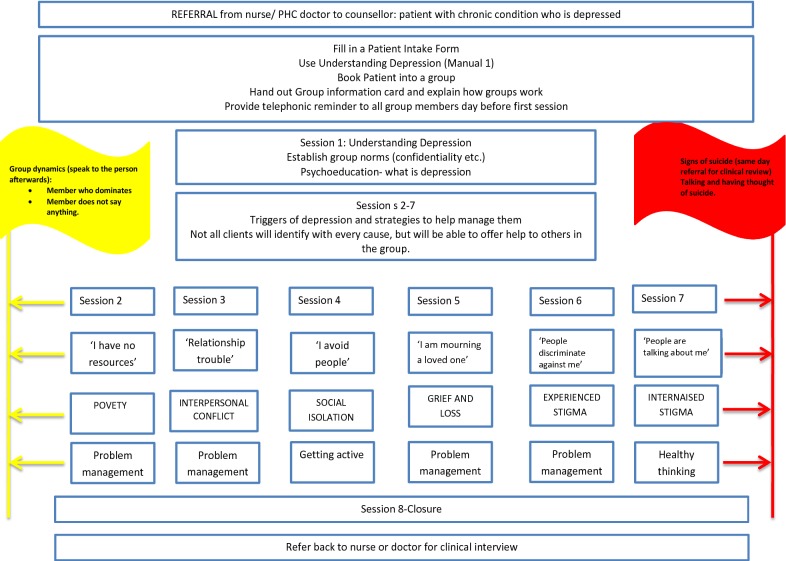
Depression intervention: sessions organogram for lay counsellors [31]

The intervention was adapted from an 8-session psychosocial depression counselling intervention developed by Petersen and colleagues in KwaZulu-Natal [9, 10] and contextualised by a formative study to understand the population sample’s experience of depression. In addition, a waiting room psychoeducational health talk was introduced, as was active follow up of service users who missed their scheduled sessions. A formative study in the study site [27] identified interpersonal conflict (including partner infidelity); grief and bereavement; experienced stigma, social isolation and perceived stigma and poverty as depression triggers and are congruent with work done previously in KwaZulu-Natal [9]. Six of the sessions were centred around each of the identified triggers and issues that maintain depressive cycles. A psychoeducation session was added to introduce service users to depression and the last session was a closure session (see Fig. 1).

In addition, a waiting room health talk on depression was introduced to help service users to identify and report their symptoms to the consulting health worker, as well as make them aware of the availability of the depression counselling service. Upon referral, on the same day, service users were taken through their first session “Understanding depression”, a psychoeducation session where the symptoms of depression were explored. The service users were given an opportunity to talk about their experience of depression, were informed of what to expect from the counselling session and how long the sessions would last. Service users were given an option to join a group or have individual counselling sessions during this session. They were then allocated to a group and given a date for the group’s first session or given a follow up date for their next individual session depending on whether they preferred group or individual sessions. The groups were sex specific, were booked over a period of 2 weeks and were formed by consecutive service users referred during this time. The size of the groups was thus affected by referral rates and ranged from a minimum of 2 service users to a maximum of 8 service users. Group and individual sessions followed a similar structure and had identical content. Group sessions were organised to include different group members’ participation. Service users who missed a group session were followed up and offered a catch-up session covering the missed session before their group’s next group session. Session attendance was voluntary and service users could drop out whenever they wanted. Service users were issued with appointment cards during the first sessions with details of their follow-up sessions, updated at the end of each subsequent session.

This counselling intervention was provided by PRIME appointed lay health workers based at the facilities, with one counsellor per facility. They had a 5-day structured group training informed by adult learning and teaching theories using experiential, interactive and reflective learning. This was followed by in vivo training and supervision of all eight sessions until competency was achieved, and regular supervision and support thereafter. In vivo supervision and training involved a supervisor assisting the lay counsellor while he/she facilitated group/individual sessions. The supervisor’s role was to model the correct way of running the session and deal with issues that may arise in the capacity of a co-facilitator so that the session itself was not disrupted. In addition, the supervisor observed how the counsellor facilitated the group and reinforced skills attained during training by providing feedback to the counsellor after the depression counselling session. The counsellor thus not only had the opportunity to observe and learn how to deal with similar issues in the future, he/she received feedback that would help with facilitation of future sessions. Supervision and support following training was developed to provide continued training and support following the apprenticeship model due to work conditions associated with managing their own emotions. It was made up of two components: group supervision and individual supervision and debriefing.

### Evaluation framework

The MRC process evaluation guide for complex interventions provided the guiding framework for the evaluation whereby implementation process indicators such as fidelity and dose of the intervention were collected alongside the cohort outcome data so as to understand implementation processes that may have impacted on the outcome findings; as well as the use of qualitative process interviews with service uses to understand mechanisms of impact in relation to participants’ responses to and interaction with the intervention [29].

### Design

A mixed method study design was adopted guided by the MRC framework described above. It included tracking key outcomes using an observational cohort study with baseline and 3- and 12-month follow-up, the collection of process indicators on uptake of the counselling intervention by patients referred for counselling, as well as follow-up qualitative interviews with referred patients. The study was conducted after PHC staff had received strengthened training to identify depressive symptoms using Adult Primary Care (APC) guidelines as part of the PRIME-SA intervention packages for depression [24] and facility-based lay counsellors had been trained in the structured manualized intervention and introduced into the clinics.

### Study population

The study population was made of adult clinic service users attending chronic care services. The inclusion criteria were adults aged 18 years and above; time and ability to complete the full interview; willingness to provide informed consent and could speak English or Setswana (the predominant language in the province). The exclusion criteria were incapacity to provide informed consent (e.g. under 18 years, presence of severe intellectual disability and/or currently experiencing an acute medical issue) assessed by the fieldworkers following training and being already on depression treatment.

The cohort sample comprised a sub-set of a larger study evaluating the impact of the introduction of the collaborative care model on both detection and service user outcomes [32]. The sub-set used in this study comprised 173 service users who were recently diagnosed with depression by a nurse or doctor and referred for the counselling intervention and no other services. Service users referred to other providers in the collaborative care model were excluded.

### Cohort recruitment

Field workers were proficient in Setswana and English and had a minimum of a grade 12 qualification. They were trained on recruitment procedures, ethical procedure, the questionnaire and use of an android device to collect data and had daily supervision from supervisors based on site. All interviews were conducted in either English or Setswana depending on the language preference of the participant.

**Fig. 2:**
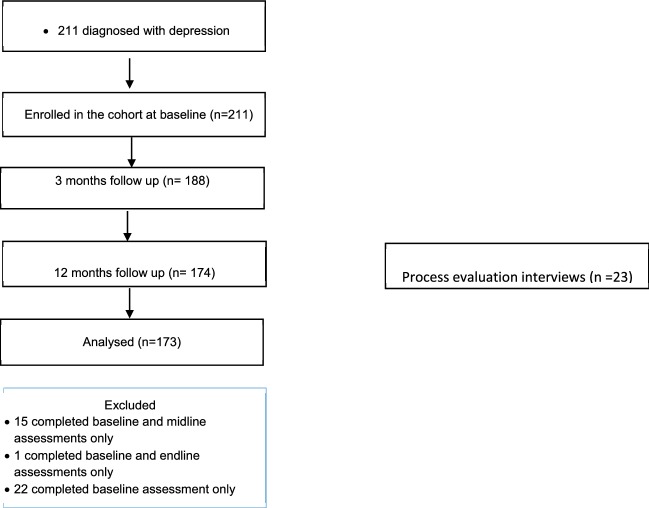
Flow diagram of study procedure for depression cohort

After the recruitment window opened, field workers approached and attempted to recruit all service users exiting from a chronic care consultation. Eligible service users were identified and recruited into the depression cohort study using informed consent procedures. Participants were administered a structured questionnaire programmed into an electronic device. The questionnaire was divided into two parts. The first part of the interview assessed service users for evidence of a diagnosis of depression upon them exiting the clinical consultation. All service users in receipt of a diagnosis of depression were enrolled into the cohort of the study and were administered the baseline interview (see flow diagram in Fig. 2) which formed the second part of the questionnaire.

### Measures for cohort study

In addition to demographic information that was collected at baseline only, the following measures were administered at recruitment (baseline) and two follow up points; midline (3 months following baseline) and end-line (12 months after baseline): The Patient Health Questionnaire 9 (PHQ9) was used to assess the impact of the depression intervention on depressive symptoms. It has been widely used in low and middle income context [33, 34] and validated in South Africa for the general population in a primary health care context [34]. The Oslo Social Support Scale (OSS-3) was used to measure perceived social support [35, 36]. The OSS-3 contains three items assessing number of close relationships; perceived level of concern from others and ease of getting help from neighbours. The higher the score the greater perceived social support. The scale has been shown to have predictive validity in relation to psychological distress and has been validated in Nigeria [37]; the World Health Organization Disability Assessment Scale 36-item (WHODAS 2.0) was used to assess subjective functional impairment in 6 domains categorised in the following ways: social functioning, self-care, mobility, interacting with other people, life activities and cognition with a recall period of 30 days [38]. The score can be calculated using simple or item-response-theory (IRT) based scoring method with higher scores indicating higher levels of disability. The scale has been used extensively around the world in different areas of research [38, 39]. It has been proven to be cross culturally viable and possesses high internal and convergent validity in primary care settings [40]. The scale has been validated in numerous studies in the American, European and Asian context. In Africa an adaptation of the WHODAS 2.0 was validated in Nigeria (12 item) [38] and the WHODAS 2.0 (36 item) was validated in Ethiopia [41]. Internalized Stigma of Mental Illness Inventory (ISMI) scale is a questionnaire made up of 29 items used to assess the impact of internalised stigma on psychiatric illness [42, 43]. The scale has been used widely in different versions and has been translated into numerous languages and has shown cross cultural reliability [42] as well as been previously used in South Africa [43].

The number of counselling sessions participants received were recorded and categorized into 0 sessions, 1–4 sessions and 5–8 sessions.

### Procedure

Service users referred for the counselling intervention and recruited into the cohort study were actively followed-up, using means which have been agreed upon as part of the informed consent process (e.g. telephoning, home visit, contact through a third party). The midline visit was set to coincide with the time-point at which the optimal effect of treatment was expected to occur and this is 3 months (± 2 weeks) after baseline. The study included participants who had received all 3 time points assessments.

The qualitative process evaluation interviews were conducted concurrently with the 12 months follow up interviews. Participants recruited into the cohort sample were asked to volunteer and were stratified by the number of counselling sessions received as follows: (1) participants who did not take up the service (0 counselling sessions; n = 7) (2) participants who received a low density dosage (1–4 counselling sessions, n = 11); and (3) those who received a high density dosage (5–8 counselling sessions; n = 5) across group and individual counselling sessions so as to understand reasons for no, poor and good uptake of the sessions. While we set out to recruit equal numbers of participants who were exposed to 0, 1–4 and 5–8 sessions, that the volunteer sampling approach resulted in variations in the number recruited for each category. Those who consented were then interviewed. The location of interviews was the primary health care facility where the participant was receiving care, or at the participant’s home where the participant could not make it to the clinic. In either location, fieldworker ensured adequate privacy for the interviews. These interviews used a semi-structured interview schedule and sought to elicit the participants’ experience of counselling intervention and why they stopped/continued with the sessions. These interviews were audio-recorded following informed consent procedures and conducted by trained research assistants conversant in English and Setswana.

### Data management and confidentiality

Handheld devices were used for data collection. Fieldworkers were trained in the use of a hand-held device to collect data, as well as in administering the survey questionnaire. The devices were programmed to facilitate the questionnaire in Setswana and in English. The data were electronically transferred to a central database, Mobenzi, through the device network for storage and analysis. Access to the data was limited to the principal investigator and two research coordinators. The data management processes (e.g. data monitoring and cleaning) were centrally managed by the PRIME management team at the University of Cape Town. The research coordinators’ computers, the Mobenzi server, the UCT server were all password-protected. No individually identifying data were centrally stored as part of research data.

### Analysis

#### Cohort study

Descriptive statistics were used to describe the cohort sample demographics. Linear regression models with number of counselling sessions and time (baseline and endline) was used as predictors, including an interaction term to establish the influence of counselling exposure on depression, functional disability, social support and internalised stigma scores between baseline and endline. Midline scores were not included in the analysis as the levels of counselling exposure utilized (0; 1–4; 5–8 sessions) overlapped with midline assessments. Post-estimation of linear combinations of coefficients at baseline and endline was used to determine differences in coefficients in relation to levels of counselling exposure at baseline and at endline.

#### Process evaluation

Interviews were translated where not conducted in English, with back translation checks applied. NVivo (version 11) was used to aid framework analysis to analyse and interpret the data collected from qualitative interviews. The process started with the author familiarising herself with the interview transcripts. A framework was developed through the aid of NVivo using a priori and emergent themes [44, 45]. The framework was refined through re-reading of the transcripts in light of emergent themes. This step overlapped with indexing where the framework was applied to each transcript while NVivo produced case charts (0 sessions, 1–4 sessions and 5–8 sessions) of the indexed data [44, 45]. The themes were first analysed for the overall sample and then particular themes that differentiated the group by dosage were looked at. The case charts varied according to different uptake of counselling sessions. The data was read both across and downwards to see what was unique to each case and how the themes looked across cases [44, 45]. The author then sought to interpret the data.

### Ethics

Ethical approval was obtained from the University of KwaZulu-Natal Biomedical Research Ethics Committee (BREC) (ethical clearance number HSS/0880/011). Field workers gave a general explanation of the study to service users in the waiting room waiting for consultation. Service users who were interested to take part were taken to a private room where a detailed explanation of the study was provided. The service users were given an opportunity to read the consent form in their preferred language (Setswana or English) and to ask questions if they had any before providing written consent and were given a duplicate of the signed consent form. Illiterate service users had the consent form read out to them verbatim in the presence of a witness who countersigned when they provided consent by marking a cross (x). Participation was voluntary and service users were informed they could withdraw from the study any time they chose to.

## Results

### Cohort study

Baseline demographics are presented in Table 1. The majority (69%, n = 120) of the sample was aged 36 years and above with women accounting for almost four-fifths (79%, n = 137) of the population, which is not unusual as South African clinics provide health services oriented to women. Just over half the population (53%) had a primary school level of education. Most people in the sample were unemployed (73.4%). Most participants (64%) received 5–8 sessions. Those who received 1–4 sessions accounted for 18.5% of the sample while 13.3% did not take up the intervention. Of those who received the intervention 58% received individual counselling while 42% received group counselling.

**Table 1:** Demographic characteristics of sample

Demographic characteristics	N = 173	(%)
Sex		
Male	36	20.81
Female	137	79.19
Age		
≤ 25	17	9.83
26–25	36	20.81
36–50	53	30.64
≥ 51	67	38.73
Education		
None	5	2.89
Non-formal	4	2.31
Less than primary school	42	24.28
Primary school	92	53.18
Secondary school	23	13.29
College/University	7	4.05
Marital status		
Single	62	35.84
Has a partner	81	46.82
Divorced/widowed	30	17.34
Employment status		
Not employed	127	73.41
Employed	46 (self, full and part-time)	26.59

Regression analysis to assess change in symptom scores by exposure category is presented below.

Regression estimates for PHQ9 (Table 2) show that participants receiving 5–8 sessions have the greatest reduction (less depression symptoms) in PHQ9 scores from baseline to endline (β = − 2.46, 95% CI − 5.06 to 0.15) compared to those with 0 sessions (β = − 0.51, 95% CI − 3.62 to 2.60, *p* = 0.064) although still not significant. A similar trend is observed in comparing sessions 1–4 to 5–8 sessions from baseline to endline.

**Table 2:** Regression estimates for PHQ9 scores in relation to counselling exposure from baseline to endline (n = 166)

Variable	Estimate	SE	z	p
0 sessions				
Baseline	11.17	1.05	10.68	0.000
Endline	8.22	1.05	7.86	0.000
Difference in PHQ9 scores	− 2.96	1.21	− 2.44	0.014
1–4 sessions				
Baseline	11.62	0.89	13.11	0.000
Endline	8.16	0.89	9.20	0.000
Difference in PHQ9 scores	− 3.47	1.02	− 3.38	0.0.001
5–8 sessions				
Baseline	12.10	0.48	25.41	0.000
Endline	6.68	0.48	14.04	0.000
Difference in PHQ9 scores	− 5.41	0.55	− 9.84	0.000
0 sessions vs. 1–4 sessions				
Endline to baseline	− 0.51	1.58	− 0.32	0.747
0 sessions vs. 5–8 sessions				
Endline to baseline	− 2.46	1.33	− 1.85	0.064
1–4 sessions vs. 5–8 sessions				
Endline to baseline	− 1.94	1.16	− 1.67	0.095

Table 3 shows the WHODAS scores decreased (better functionality) significantly more from baseline to endline among those who received 5–8 sessions (β = − 10.73, 95% CI − 19.86 to 1.59) compared to those with 0 sessions (β = 2.25, 95% CI − 8.65 to 13.14, *p *= 0.021). An even greater decline in scores was noted from baseline to endline among those who received 5–8 sessions compared to those who received 1–4 sessions.

**Table 3:** Estimation of differences in WHODAS scores in relation to counselling exposure from baseline to endline (n = 166)

Variable	Estimate	SE	z	p
0 sessions				
Baseline	30.80	4.16	7.40	0.000
Endline	28.99	4.16	6.97	0.000
Difference in WHODAS scores	− 1.82	4.24	− 0.43	0.669
1–4 sessions				
Baseline	35.16	3.53	9.97	0.000
Endline	35.59	3.53	10.09	0.000
Difference in WHODAS scores	0.43	3,60	0.12	0.904
5–8 sessions				
Baseline	34.58	1.89	18.26	0.000
Endline	22.05	1.89	11.64	0.000
Difference in WHODAS scores	− 12.54	1.93	− 6.49	0.000
0 sessions vs. 1–4 sessions				
Endline to baseline	2.25	5.56	0.40	0.686
0 sessions vs. 5–8 sessions				
Endline to baseline	− 10.73	4.67	− 2.30	0.021
1–4 sessions vs. 5–8 sessions				
Endline to baseline	− 12.97	4.08	− 3.18	0.001

While the ISMI scores show an overall significant decline (less stigma) in scores at endline (Wald X^2^ = 61.60, *p *= 0.000), no significance was noted as a function of levels of counselling exposure from baseline to endline among those who received 5–8 sessions (β = − 1.80, 95% CI − 5.51 to 1.91) compared to those with 0 sessions (β = 2.25, 95% CI − 2.18 to 6.67, *p *= 0.341) but was significant for those who received 5–8 sessions compared to those who received 1–4 sessions (β = − 4.05, 95% CI − 7.303 to − 0.800, *p* = 0.015) (Table 4).

**Table 4:** Estimation of differences in ISMI scores in relation to counselling exposure from baseline to endline

Variable	Estimate	SE	z	p
0 sessions				
Baseline	27.09	1.65	16.46	0.000
Endline	23.09	1.65	14.03	0.000
Difference in ISMI scores	− 4.00	1.72	− 2.32	0.020
1–4 sessions				
Baseline	26.53	1.39	19.02	0.000
Endline	24.78	1.39	17.76	0.000
Difference in ISMI scores	− 1.75	1.46	− 1.20	0.231
5–8 sessions				
Baseline	28.13	0.75	37.56	0.000
Endline	22.33	0.75	29.81	0.000
Difference in ISMI scores	− 5.80	0.75	− 7.39	0.000
0 sessions vs. 1–4 sessions				
Endline to baseline	2.25	2.26	1.00	0.319
0 sessions vs. 5–8 sessions				
Endline to baseline	− 1.80	1.89	− 0.95	0.341
1–4 sessions vs. 5–8 sessions				
Endline to baseline	− 4.05	1.66	− 2.44	0.015

The OSS-3 scores failed to show an overall significant difference (Wald Χ^2^ = 3.58, *p *= 0.61) with no significant difference as a function of levels of counselling exposure from baseline to endline among those who received 5–8 sessions compared to those with 0 sessions, as well those who received 1–4 sessions (Table 5).

**Table 5:** Estimation of differences in OSS-3 scores in relation to counselling exposure from baseline to endline

Variable	Estimate	SE	z	p
0 sessions				
Baseline	8.09	0.48	16.94	0.000
Endline	8.52	0.48	17.85	0.000
Difference in OSS-3 scores	0.43	0.55	0.79	0.430
1–4 sessions				
Baseline	8.53	0.40	21.07	0.000
Endline	8.81	0.40	21.77	0.000
Difference in OSS-3 scores	0.28	0.47	0.60	0.547
5–8 sessions				
Baseline	8.98	0.22	41.32	0.000
Endline	8.94	0.22	41.12	0.000
Difference in OSS-3 scores	− 0.04	0.25	− 0.18	0.857
0 sessions vs. 1–4 sessions				
Endline to baseline	− 0.15	0.72	− 0.21	0.832
0 sessions vs. 5–8 sessions				
Endline to baseline	− 0.48	0.60	− 0.79	0.428
1–4 sessions vs. 5–8 sessions				
Endline to baseline	− 0.33	0.53	− 0.62	0.538

### Process evaluation

A summary of the process evaluation themes is presented in Table 6. Overall, 14 out of 16 service users who received the intervention reported it helped with symptom improvement including insomnia, social isolation, appetite, irritability and also provided someone to confide in.

It is not the same as before now I have an appetite although I still think a lot it is not the same […] I could not [cook or do laundry] before as I wanted to do nothing when I woke up in the morning all I wanted to do was to just sit. [I: Mmm] So now I have the strength to do it I wake up in the morning and do everything that I am supposed to do (*Participant 14; 1*–*4 sessions*)

**Table 6:** Themes summary

Themes	Counselling sessions		
	0	1 to 4	5 to 8
Referral process and intervention uptake How the referral process was handled was linked to the number of counselling sessions received	Did not take up the intervention because they didn’t know what to expect Did not meet with the counsellor on day of referral Had other pressing things to attend to	The referral process was not explained for four of the participants while four participants asked to be referred to the counsellor	Four of the five service users interviewed reported having the referral explained to them and informed on what to expect from the counselling sessions Introduced or walked to the counsellor
Benefits of enhanced dosage			Reported improved interpersonal relationships, being more socially active, reduced internalised stigma, improved sleep and increased self-confidence Improved self-reliance and self-efficacy
Design of the counselling manual	Helped service users feel safe and not exposed while sharing their experiences Vignettes presented depression factors in a way that was relatable for the service users		
Reasons for dropping out and not taking up intervention	Not knowing what to expect from the counselling intervention Contextual factors	Feeling better Ill health Job opportunities and Contextual factors	
Motivation to continue with sessions		Self-observed improvement of symptoms provided an incentive for participants with expectation for further improvement	Self-observed improvement of symptoms provided an incentive for participants with expectation for further improvement Counsellor qualities: as empathetic, able to contain participants’ emotions, explained structure of the intervention, displayed competency, set appointments and followed up service users

#### Intervention uptake

The referral process was reported to play a key role in uptake of intervention. Three aspects of referral were identified by service users namely diagnosis, referral explanation and connecting participant to counsellor. Four of the five service users interviewed who had received 5–8 sessions reported having the referral explained to them and informed on what to expect from the counselling sessions. Some service users in this group (2 out of 5) reported being walked over to the counsellor’s room or getting introduced to the counsellor after being diagnosed/identified with depression.

She […] gave me a paper and told me to go to [the lay counsellor] I did not know who [the counsellor] was and she took me to her […] (*Participant 22; 5*–*8 sessions*)

Yes, she [explained the referral process]. I wouldn’t have gone anywhere if she didn’t. (*Participant 24; 5*–*8 sessions*)

The referral pathways were, on the most part, not clear nor was the process explained to the service users. Service users, particularly those who did not take up the intervention and those who received 1–4 sessions, reported not understanding why they were referred, who they were referred to, and how that person would help them. Almost all service users who did not take up the intervention and some who received 1–4 counselling sessions reported not understanding the referral and a vagueness about what was to follow.

I was very nervous, not knowing the outcome got me feeling scared. (*Participant 9; 1*–*4 sessions*)

Lack of clarity on referrals, leading to not knowing what to expect from the counselling service after the referral was made led to some service users not taking up the intervention.

I am at fault because I did not come and I promised them (I: Mmm) I did not know what was going to happen (I: Mmm) That is the reason - I do not want to lie. (*Participant 1; 0 sessions*)

Other reasons that led to service users not taking up the intervention when referred included not meeting with the counsellor after being referred, home responsibilities, job opportunities and not having set appointments. One participant explained that she could not take up the services because she was called away to look after her ailing sister who then passed away after some time. She then had to help out with funeral procedures after her sister passed away and to be around her family after the funeral. Another’s time was taken up by caring for a young child and making herself available for ‘piece job’ opportunities.

#### Benefits of enhanced dosage

Improved dosage of the counselling intervention was affected by service users’ experience of the intervention and counsellor qualities. Experience of intervention was informed by the service users’ awareness of psychosocial change, structure of the sessions, counselling groups’ dynamics and intervention material. Service users who received 5–8 sessions reported improved interpersonal relationships, being more socially active, reduced internalised stigma, improved sleep and increased self-confidence.

Alright, the sessions really changed me a lot the first time when I attended the session I would shout (at) my children at home for nothing and I would feel hurt, now it has brought back my life because I would sleep the whole time and would not cook and eat, I would not wash the dishes living in unkempt conditions but now it was helped me a lot as I do not do those things anymore and I do not undermine myself […] (*Participant 22; 5*–*8 sessions*)

#### Improved self-reliance and self-efficacy

The intervention reportedly helped in promoting greater self-reliance and self-efficacy. Service users who received 5–8 sessions also reported the intervention empowered them (3 out of 5) to deal with poverty through improved budgeting and identifying income generating opportunities as ability to manage identified problems.

I would say the sessions made that possible, because it helped me to want to get up and do something with my life, like selling achar for income, and not just sitting waiting on my husband. […] right now, I’m making means to get extra money for the household, when my husband can’t buy some things I can use some of the money to supplement. (*Participant 19; 5*–*8 sessions*).

I can now talk [about my condition] I have informed my children about my status […] when I came I told [the counsellor] I want to tell my children that I am HIV positive […] I was afraid of telling them but I ended up telling them and they accepted me […] I now don’t hide when I take my medication […] (*Participant* 22, *5*–*8 sessions*)

#### Design of the counselling manual

The depression counselling manual and sessions were designed and structured (using vignettes and protagonists) so as to help service users feel safe and not exposed while sharing their experiences. Identifying with the protagonist’s experience of the depression-stress-factor of the day allowed for the group to discuss the issue in a safe way. The vignettes presented depression factors in a way that was relatable for the service users.

Yes, it was easy for me to accept because as the scenarios were being read, I could relate them to my personal experiences. […] (Participant 24; 5–8 sessions)

The vignettes enabled the service users to talk about their lived experience of depression and they were thought to illustrate the truth as the stories captured the reality of their lived lives.

[Thandi’s story] is the one that made us talk about life […] It had an impact because I had similarities with her (*Participant 16; 1*–*4 sessions;* emphasis added).

#### Reasons for dropping out

At least 2 people who had received 1–4 sessions reported they did not come for follow up because they were feeling better. Other reasons for dropping out of the intervention include ill health; job opportunities and contextual factors. Participant 10, who was 76 years indicated that she was not able to make the weekly sessions because of ill health. *Participant 8* could not continue with the intervention because she was taking care of her wheelchair bound husband and a young child giving her little time to for anything else.

I have to look after my husband as he uses a wheelchair and the baby so at times the time that’s set which is mostly 8am to 9am I don’t have time because I have to prepare for the people going to school first then I have to attend to my husband and I have a baby on the other side so it’s just lack of time for me (*Participant 8; 1*–*4 sessions*)

At least 2 of the service users were required to visit their traditional home for a prolonged time and could not continue with the sessions. Participant 13 put her sessions on hold to be resumed at a later stage to go for traditional healing initiation.

#### Motivation to continue with sessions

Self-observed improvement of symptoms provided an incentive for participants (4 out 5 participants who attended 5–8 counselling sessions; 2 who attended 1–2 sessions) to continue with the counselling sessions with expectation for further improvement.

I saw that going for counselling will help me in a lot of ways [compared to what] I had received before. (*Participant 17; 1*–*4 sessions*)

Counsellor qualities played a role in encouraging participants to attend counselling sessions. Counsellors who had service users with the most sessions were described as empathetic, able to contain participants’ emotions, explained structure of the intervention, displayed competency, set appointments and followed up service users. The counsellor providing information on how the service was organised seemed to help with attendance as the participants reportedly knew what to expect. For participants who received 5–8 counselling sessions, 4 of the 5 reported receiving the information compared to 1 out of 11 participants who were exposed to 1–4 sessions. The counsellor also made follow-ups and was able to establish rapport with service users enabling some of them to take responsibility for their appointments. Although the counsellor was viewed as young and inexperienced by older service users, her facilitation skills helped with gaining the service users’ trust. She was also credited with creating a safe space where service users felt safe to talk about their experiences, identifying with the protagonist in the stories but not feeling exposed.

[[The counsellor] is good. She does her job with integrity without discrimination […] she is not the type of person that judges people; she just really knows how to talk to people, with everyone […] she [made telephone reminders], but then I already knew when to attend my sessions, if I had an issue with attending I’d phone to alert her. (*Participant 18; 5*–*8 sessions*)

## Discussion of results

Guided by the Medical Research Council (MRC) framework for the evaluation of complex interventions, this study had two objectives: (i) to evaluate the relationship between levels of exposure to the task-shared counselling intervention component of the collaborative care model, and psychosocial outcomes (depression, functional disability and social support) in chronic care service users with comorbid depression; and (ii) to understand implementation and participant-level mechanisms of impact that promoted greater exposure to the intervention guided by the MRC framework for process evaluation. The majority of the sample enrolled in the study were women, a demographic feature characteristic of primary health facilities providing chronic care in South Africa [46–48].

The intervention led to a clinically significant reduction of depression symptoms at 12 months follow up. Service users who were exposed to 5–8 counselling sessions showed greater reduction in PHQ9 scores (although still not significant) compared to those who had no exposure to the intervention. This outcome corroborates international findings of similar studies with lay counsellor led interventions [26] and adds to the evidence in Southern Africa that adapted behavioural cognitive techniques can be successfully delivered by non-specialists [10, 27, 49, 50].

Participants who received of 5–8 sessions had better PHQ 9 outcomes at 12 months compared to those having zero or 1–4 sessions, suggesting 5–8 sessions to be the optimal dosage. It is not clear why service users who did not take up the intervention had similar PHQ9 outcomes to those who received 1 to 4 counselling sessions. One explanation could be service users with 0 counselling sessions did not, in-fact, need the service while those with 1 to 4 counselling sessions needed to continue with the counselling sessions. More robust methods are needed to investigate this further. Qualitative interviews with participants who received 5–8 sessions suggests that more sessions helped to promote greater attainment of skills to help negotiate life circumstances better. This finding is supportive of the NICE guidelines based on evidence from mostly high-income countries that recommends 6 to 8 sessions for low-intensity psychosocial intervention as optimal [51].

Evidence shows that functional disability is strongly associated with depression [52–55]. While there was no significant difference between baseline and endline WHODAS scores for the entire cohort, functional disability decreased significantly from baseline to midline among those who received 5–8 sessions. This suggests that 5–8 sessions are required for counselling to have an impact on improving functional ability. These functional improvements are supported by the qualitative process evaluation interviews, where participants who attended 5–8 counselling sessions reported an improvement in self-reliance and self-efficacy following receipt of the intervention. They reported that the counselling sessions capacitated them to carry out a plan to reach a desired goal which has been associated with enhanced empowerment or self-efficacy [56, 57]. These capacities are essential for self-management which has been shown to yield good results in chronic diseases management within the collaborative care model [57, 58].

Exposure to more sessions also produced better benefits for self-stigma. There was a significant difference in reduction in internalized stigma between participants who attended 1–4 sessions compared to those who attended 5–8 sessions at endline. The fact that the session on internalized stigma was generally delivered later in the programme could potentially explain this finding. It is not clear why service users who did not take up the intervention had better self-stigma outcomes than those who received 1 to 4 counselling sessions. As with the PHQ9 outcomes it could be service users with 0 counselling sessions did not need the service while those with 1 to 4 counselling sessions needed to continue with the counselling sessions. More robust methods are needed to further investigate this. Self-stigma has a negative impact on self-esteem and self-efficacy and is considered a risk factor for poor mental health [56, 59]. Literature also shows that higher levels of self-stigma are associated with higher levels of depression [42].

In relation to social support, the overall OSS-3 mean scores showed no significant improvement for perceived social support. This is similar to Petersen et al.’s [10] findings where there was no improvement in perceived social support for a group psychosocial intervention led by lay counsellors even though the buffering effect of perceived social support has been associated with improved mental health [35, 36]. OSS-3 has not been validated for the South African context and hence it is possibly a function of the measure used and worth exploring further.

In relation to reasons for greater counselling attendance, the qualitative process evaluation interviews revealed that availability of services, awareness of helpfulness of counselling sessions and counsellor qualities helped with attending more counselling session. It appears having insight, the process of becoming increasingly aware of one’s mental state [60], promoted adherence to the intervention. The counsellors, especially those for participants who attended 5–8 sessions, were also reported as having more person-centred qualities that promoted trust, understanding and a safe counselling space and allowed for the participants to self-express. Person-centred counselling promotes service user’s self-expression, self-awareness and a self-understanding and helps facilitates self-management [61].

In addition, the referral process emerged as important in promoting greater uptake and adherence to the counselling sessions. Service users who had received 5–8 sessions reported that the referring nurse accompanied them to the counselling room or introduced them to the counsellor following the referral. This group also reported meeting with the counsellor at set times. In contrast, participants who did not take up the service and those who attended 1–4 sessions reported unexplained referral process, contextual issues making it difficult to attend counselling sessions, inconsistent appointments and not meeting with the counsellor on the day the referral was made. Linking service users with service providers can help with improving access to health services [62]. From this study, it is clear that in the context of low mental health literacy, there is a need for mental health referrals to be explained and having on-site referral resources elevated.

Feeling better, contextual issues including having a day job, caring for sick family members and helping with funeral arrangements were reasons reported for dropping out before completion of counselling sessions. This tended to affect female service users who played the role of primary care giver more. Not only were their services seen as expendable, they were not able to attend to their own health care needs, with the needs of other family members outweighing their own.

## Conclusion and recommendations

The findings of this evaluation of a lay-counsellor delivered psychosocial counselling intervention for chronic service users found that it helped reduce depression symptoms, at 12 months follow-up; with 5–8 sessions found to be optimal in this regard. Participants receiving this optimal number of sessions displayed improved functionality and reduced self-stigma, reported to have been empowered to self-manage, be more self-reliant, and capacitated with skills to improve their quality of life. Factors found to optimize attendance of counselling sessions included counsellors with person-centred care qualities, referral processes that included being provided with an explanation as to how counselling could benefit the service user by the referring nurse, and being connected to the counsellor either by being walked to the counselling room or introduced to the counsellor.

## Data Availability

The anonymised data will be made publicly available, in accordance with PRIME publication and data management policies available at https://bit.ly/2tXQQsV.

## Abbreviations

NCDs: non-communicable diseases
CMDs: common mental disorders
PHQ9: Patient Health Questionnaire
ART: antiretroviral treatment
HIV: human immunodeficiency virus
AIDS: acquired immune deficiency syndrome
DALYs: disability-adjusted life year
WHO: World Health Organisation
PRIME-SA: Programme for Improving Mental health CarE South Africa
CBT: cognitive behavioural therapy
mhGAP: Mental Health Gap Action Programme
APC: adult primary care
OSS-3: Oslo Social Support Scale
WHODAS 2.0: World Health Organization Disability Assessment Scale 36-item
ISMI: Internalised Stigma of Mental Illness Inventory
UCT: University of Cape Town
BREC: Biomedical Research Ethics Committee
